# Multi-Farm Analyses Indicate a Novel Boar Pheromone Improves Sow Reproductive Performance

**DOI:** 10.3390/ani9020037

**Published:** 2019-01-27

**Authors:** John J. McGlone, Arlene Garcia, Anoosh Rakhshandeh

**Affiliations:** 1 Laboratory of Animal Behavior, Physiology and Welfare, Department of Animal and Food Sciences, Texas Tech University, Lubbock, TX 79409, USA; arlene.garcia@ttu.edu; 2 Animal Biotech, Dallas, TX 75201, USA; 3 Department of Animal and Food Sciences, Texas Tech University, Lubbock, TX 79409, USA; Anoosh.Rakhshandeh@ttu.edu

**Keywords:** pigs, sow, pheromone, reproductive performance

## Abstract

**Simple Summary:**

Pork production farms are more sustainable if the total number of pigs per batch is increased with a given sow herd size. Improving breeding and farrowing rates as well as litter size will increase farm output per sow. This field study examined the effect of application of a novel boar pheromone (BOARBETTER^®^; BB) containing three active molecules. Effects on sow reproductive efficiency were measured as breeding and farrowing rates, pigs born alive, stillborn and total numbers born. Farms applied BB in alternating weeks. Among records from nearly 4000 sows enrolled in the study, the use of BB increased the number of pigs born alive per litter and total pigs born per farrowing batch. BOARBETTER^®^ is a cost effective and safe product that has the potential to meaningfully improve sow reproductive performance.

**Abstract:**

The objective of this study was to examine the effectiveness of a novel 3-molecule boar pheromone (BOARBETTER^®^, BB,) to improve sow reproductive performance (breeding, conception, farrowing rates, pigs born alive, stillborn, mummies and total born). Data from 12 commercial farm sites were used to evaluate the effectiveness of BB. Each farm was used as the experimental unit in the meta-analyses. Individual sows records were collected, merged and analyzed in overall analyses. Relative to CON, BB increased the number of total born pigs per litter (13.81 ± 0.11 vs. 14.30 ± 0.11 pigs/litter, respectively; *p* < 0.01) and the number of pigs born alive (12.76 ± 0.14 vs. 13.13 ± 0.14 pigs/litter, respectively; *p* < 0.05). In the merged dataset analyses, the parity by treatment interaction was significant for total pigs and pigs born alive per litter (*p* < 0.01). In parities one through three, treatment with BB increased total pigs born by 0.88 per litter, and pigs born alive per litter by 0.73 pigs per litter (*p* < 0.05). However, BB had no effect on these parameters in sows from parities four through six. BOARBETTER^®^ increased reproductive success, is cost effective, safe, and can meaningfully improve sow reproductive success and performance.

## 1. Introduction

Pork production around the world depends on identifying sows in estrus, successful mating and farrowing large litters of pigs per litter. The total number of pigs born and weaned in a group is a function of both breeding/farrowing rates and number of pigs per litter. Boar and semen management improve reproduction, however, sow management is also an area in which commercial pork production can be improved. Litter size has increased in recent years through genetic selection and improved housing and management, though breeding and farrowing success rates remain a challenge for the swine industry [1].

Any tools that might improve farrowing rates and litter size would improve semen and mating quality to attain improved reproductive performance. Farrowing rate does not respond well to genetic selection [2]. Thus, genetic improvement has focused on increasing the numbers of pigs born alive per litter. Approximately, 90% of sows express estrus between 3 and 6 days after weaning [3,4]. Mating during estrus leads to greater farrowing rates and litter sizes than sows bred later than 6 days after weaning [2]. Failure to detect estrus greatly affects farrowing rates and litter size [5]. 

Pigs are among the species with the greatest number of functional olfactory receptors [6]. Biologically relevant olfactory signals can change the brain, physiology and behavior in important ways. Despite its large olfactory acuity, little direct attention has been spent on commercial farms to study or manage the olfactory environment. Many producers use teaser boars to help gilts develop and to identify sows in estrus. Teaser boars are used in part to spread pheromones near gilts and sows. Use of live adult males would deliver variable concentrations of pheromones, but they also provide visual, auditory and at time tactile signals to the females.

The effectiveness of pheromones has been discussed in the scientific literature since the 1950s. In the 1960s, Patterson [7,8] reported that boar saliva contained 2 steroids that act as boar pheromones that stimulate sow reproduction. In behavioral tests, these steroids improved the percentage of weaned sows or gilts that express sexual behaviors, but not as much as a live boar [9]. The single odorous molecule androstenone has been sold as a commercial product to stimulate sow behavior and reproduction for several decades.

Knowing that androstenone causes an incomplete sexual behavioral response [9] in weaned sows caused us to search for additional molecules that might make a more effective boar pheromone. We recently reported that a mixture of androstenone, androstenol and quinoline induced sexual behaviors in weaned sows. A commercial product was patented [10] and formulated by Animal Biotech with the name BOARBETTER^®^ (BB). The objective of this study was to examine the effects of BB, a novel boar pheromone-based product, applied on commercial farms as a potential method of increasing sow reproductive performance. We hypothesized that use of a complete boar pheromone would stimulate sow behavior, physiology and reproductive success.

## 2. Materials and Methods

### 2.1. General Methods and Treatments

All methods and procedures for this experiment were reviewed and approved by the Texas Tech University (TTU) Animal Use and Care Committee (ACUC approval number; 15038-05). All the work was performed on commercial swine farms.

A total of 3998 sows from twelve farm sites were included in this study. The twelve farm sites had sow inventories ranging from approximately 450 to 6000 sows. Farm sites were in the USA states of Arkansas, Iowa, Illinois, Indiana, Oklahoma and Minnesota. A farm site was defined as a sow unit with a certain manager that collected data in a reliable fashion as a unit. The sites had 8 owners (some owners enrolled 1 and others enrolled 2 farm sites in the study) and represented eight farm systems or owners. For enrolled farms that had two sites, one site served as a control (CON), while the other served as a BB site. For all other farm sites, treatments were alternated over 4 to 7 weeks, applying CON or BB on alternate weeks. The study was started in the month of April through October, 2017 and litters were born between August, 2017 and January, 2018.

Sows were housed in farm specific breeding areas, consisting mainly of breeding crates. Sows were assigned each treatment (Con or BB) by breeding group because pheromones could not be effectively applied to adjacent individual animals without cross-contaminating neighboring sows. On all farm sites, the farm staff applied BB and collected production data following training by Animal Biotech (ABT) personnel. For farm sites 1 and 2 farm staff applied the BB product on day 4 and 5 after weaning. All other sites applied BB on days 4 and 5 unless a sow was not bred on day 4, in which case the farms continued applying BB up to 7 days after weaning. If a sow was not bred by day 7 after weaning, she was considered not bred in the study time frame.

Farm staff applied nothing during the control weeks. We have evidence from other studies [10] that BB initiated sexual behaviors relative to a vehicle control. Because this is a field study, and because pork producers normally do not spray anything on sows prior to mating, we used the appropriate field control of spraying nothing for the CON treatment group. Farms otherwise followed their normal breeding procedures during control and BB weeks. Sows on the CON treatment were bred following standard farm procedures for that owner. When BB was applied, sows received BB directed to the nose of individual sows using a garden-type sprayer for approximately 1 second (to administer 4 mL of spray). The distance of the spray was from approximately 10 cm and was sprayed prior to estrus detection with a boar. One farm site (site 10) used post-cervical insemination (PCAI) and boars were not present during breeding. Data from this farm were excluded from analyses, however the data are reported in the text of the results. All other farm sites used conventional artificial insemination with a boar present during breeding. On farm site 5, the farm analyzed the data internally and reported the means to the authors which could be included in the meta-analyses. Thus, farm 5 data are included in the meta-analyses and not in the individual sow data. Weaned sows were removed from the study if they served as nurse sows, as they typically had a longer lactation period. Sows with a lactation length of less than 17 days were excluded from the study due to inherent reproductive problems in these sows. These early-weaned sows were weaned early due to a health problem with the sow or piglets. Sows that were bred prior to 4 days post-weaning were omitted because application started on day 4 post-weaning. Sows culled after breeding but prior to farrowing were not omitted from the breeding analyses—these sows often aborted or had a health issue.

### 2.2. Observations and Calculation

Definitions of response variables are given in Table 1. Two measures not commonly reported are conception success rate and farrowing success rate

Conception success rate and farrowing success rate (FSR) were calculated as the number of sows conceived or farrowed relative to the number of sows weaned and eligible to be bred (i.e., not culled or intentionally not mated). Typically, conception and farrowing rates are determined relative to the sows bred (not relative to those eligible to be bred).

Litter size measures included piglets born alive, stillborn or mummified piglets. Total pigs born was the sum of stillborn and pigs born alive. Some farms reported only total pigs born because they believed that they could not determine if a piglet was stillborn or born alive and dies shortly after birth. Other farms reported pigs born alive or stillborn.

### 2.3. Statistical Analysis

Data were normally distributed and tested for assumptions of parametric analyses using the Univariate procedure of SAS (Cary, NC, USA). The distributions of reproductive performance variables were not skewed, nor did they show signs of Kurtosis. The mean and medians were similar as well.

Three analyses were performed. First, within-farm analyses were calculated using the sow as the experimental unit. A power test was calculated using R software for the key measure of pigs born alive per litter. The overall standard deviation (3.44) was used for control born alive. Alpha was set at 0.05 and power (1-beta) at 80% to estimate the needed sample size (number of litters) to detect a difference of 0.4 pigs born alive per litter. Because few farm sites had enough numbers of sows, these analyses were provided for descriptive purposes. Two more powerful analyses were performed, including individual sow analyses with the merged dataset and meta-analyses. Regulatory agencies in the USA ask for meta-analyses whereas other countries may prefer analyses of the merged dataset. Therefore, data were analyzed both ways.

In the meta-analyses, each farm site provided a single data point that was the Least Squares means (LSM) of each response variable. The meta-analyses had 8 to 11 farm sites per measure. Some farms did not report some measures. The farm was the experimental unit for the meta-analyses. While each farm site mean had different numbers of sow observations per measure, the meta-analyses weighs each variable on each farm equally. To obtain farm average values for each measure, LSM were calculated for each measure for CON and BB groups at each farm site using SAS (SAS version 9.4, Institute Cary, NC, USA). 

In a third set of analyses, the individual sow data from all available farms was subjected to statistical analysis using MIXED models (PROC MIXED within SAS). In these analyses farms with more sows had more weight in the overall treatment LSM. Available individual sow/litter data were included in an overall dataset that included over 3500 sows bred and over 2500 from which litter data were available. These overall analyses included only parities 1 through 6 because of very low sample sizes on some farm sites for higher parity sows (over 6). Statistical analysis of data was carried out using MIXED procedure in SAS (version 9.4, Institute Cary, NC). Data were analyzed using a completely randomized design (CRD) with treatment, parity, interaction between treatment and parity, and site as fixed effects, and sow within site as subject effect. An appropriate covariance structure was selected for analyses by fitting the model with the structure, which provided the ‘best’ fit, based on Akaike information criterion (AIC) and Schwarz Bayesian criterion (BIC). Previous lactation length was tested as a co-variable and if not significant, a reduced model was used. In this overall dataset, the covariates tested were not significant (*p* < 0.10). Values are reported as LSM ± SE. Means were separated using the Tukey–Kramer test. Treatment effects were considered significant at *p* ≤ 0.05. A tendency towards a significant difference between treatments means was also considered at *p* ≤ 0.10. Some farms did not report stillbirths or mummies and some farms had no data for certain parities. 

## 3. Results

The measures of reproductive success are listed on Table 2 from the within-study analyses and the overall meta-analyses. The total number of pigs born per litter was higher for sows treated with BB compared to CON sows, 14.21 ± 0.11 compared to 13.75 ± 0.11 pigs per litter, respectively (*p* < 0.01). Pigs born alive per litter was also higher for sows treated with BB than for CON sows, 13.01 ± 0.16 compared to 12.75 ± 0.14, respectively (*p* = 0.05). Although not significant, farrowing success rate was 3.3% higher among BB-treated sows compared with the Control sows (*p* = 0.076). One farm that used post-cervical artificial insemination (PCAI), had a 0.85 ±0.20 increase (*p* < 0.01 with in pigs per litter increase (*p* < 0.01)

Individual farm average data for each farm site are reported in Table 3. The *p*-values represent the within-farm analysis of variance (ANOVA) that examined only treatment effects. Overall, BB tended to increase the total number of pigs born compared to the CON on farms 10 (15.18 ± 0.19 compared to 14.33 ± 0.21, respectively) and 12 (16.10 ± 0.37 compared to 15.19 ± 0.37, respectively). Additionally, BB significantly increased the number of pigs born alive on farm 10 compared to control (14.13 ± 0.18 and 13.52 ± 0.20, respectively). 

The treatment effect was examined within each parity because the overall treatment by parity interaction was highly significant (*p* < 0.0001). BOARBETTER increased the total number of pigs born compared to CON in parities 1 (13.9 ± 0.23 and 13.1 ± 0.25, respectively), 2 (14.8 ± 0.20 and 13.9 ± 0.19, respectively) and 3 (15.4 ± 0.19 and 14.5 ± 0.20, respectively; *p* < 0.05). BOARBETTER^®^ increased pigs born alive per litter compared to CON for parities 1 (13.1 ± 0.23 and 12.4 ± 0.24, respectively), 2 (13.8 ± 0.19 and 13.0 ± 0.18, respectively) and 3 (14.2 ± 0.16 and 13.5 ± 0.18, respectively; *p* < 0.05). However, for parities 4, 5 and 6, BB and CON treatment groups did not differ. In parities 1–3 the average increase in total pigs born was 0.88 per litter and pigs born alive was 0.73 pigs per litter (*p* < 0.01). The power test revealed that based on the variation observed in the field, to detect a difference of 0.4 pigs born alive per litter (SD = 3.44; alpha = 0.05; power = 80%) requires 588 litters per treatment.

## 4. Discussion

### 4.1. BOARBETTER^®^ Effects on Sow Reproductive Performance

The average farm response overall is provided in Table 2. These means are obtained by averaging data from average farm sites in Table 3. Providing the farm-site averages allows the reader to take farms out and re-calculate the average response or to weight farms differently if they wish. 

Maintaining non-pregnant, non-lactating sows is expensive and contributes to the inefficiency of sow reproduction. In the U.S.A. estimates suggest the farrowing rate is about 83% [5]. The farrowing rate does not reflect all the inefficiencies in sow reproduction. The farrowing rate is typically calculated as the number of sows that farrow relative to the number that were bred. If one considers the number that farrow relative to those sows eligible to be bred (weaned sows not culled), then the farrowing success rate is lower than 83%. We included both the breeding rate and the farrowing rate when calculating the farrowing success rate (number farrowed relative to number eligible to be bred).

Season, nutrition, disease, embryo mortality before 30 days of pregnancy, and piglet pre-weaning mortality are all factors that prevent the potential for an increase in weaned pigs/mated/sow/year [5]. The total numbers of pigs born or weaned per litter in each batch of sows depends on both breeding efficiency and litter size. Increasing either or both the numbers of sows farrowing or the numbers of pigs per litter will increase the output of a given batch of sows.

We can estimate the total pigs born alive per batch for BB and CON treatments. Per 100 sows farrowing in a model control farm, use of BB would result in 103.7 litters farrowing (based on 3.7% higher farrowing success rate). Litter size born alive increased from 13.0 to 13.7 on control vs BB farms among parity one through three sows. Thus, a control farm model of 100 sows would have 1300 piglets born alive. Using BB, 103.7 litters times 13.7 pigs born alive would yield an estimated 1420 piglets born alive. This represents a potential 9.3% more piglets born alive in each batch of pigs when BB was used, based on average farm data (Table 4). To our knowledge, this is the first report documenting an improvement in sow reproductive performance by use of a pheromone in a commercial setting.

### 4.2. Analyses of Reproductive Performance

To demonstrate efficacy of any reproductive technology, one needs a very large sample size to detect economically and biologically meaningful differences. A small difference in litter size, for example, can have a large economic impact. From our data, we calculated a power test to determine the number of litters required to show a significant (*p* < 0.05) difference in litter size when the treatment means differ by 0.4 piglets born per litter (see Results for details). We determined that one needs 588 litters per treatment (or 1176 litters if two treatments were examined). No university or government research farm has been identified with enough numbers of available sows to make this comparison without confounding issues of variable performance over time. Likewise, even using commercial farms, few farms can organize over 1000 litters for a single controlled study. Thus, several commercial farm sites were used to get enough sample size to address the central hypothesis that BB might positively impact sow reproductive performance. Some scientists prefer to use the farm as the experimental unit while others prefer to use the sow as the experimental unit. The idea was to conduct the study on a large scale so that either method of analysis could be performed. If both analyses yielded a similar outcome, then one would have confidence that the differences were biologically and economically meaningful.

We examined the effects of BB on reproductive performance using two methods of analyses. In the meta-analyses, each farm has a single value in the analyses; thus, the farm is the experimental unit. This method gives equal weight to farms with small or large numbers of observations. In a second set of analyses, all the data were merged into single analyses with the sow as the experimental unit, but accounting for variation in farm sites and sow parity. The average farm data for total born and born alive per litter are given in Table 3 while the averages of these measures are given in Table 2. The average farm data are provided so the reader can remove single farms and re-calculate the average response. For example, if one removes farm 10 from the analysis (because this was the only farm that used PCAI) for total born, the average changes 0.49 to 0.46 more pigs per litter born. Removing single farms from the analysis while maintaining the general response speaks to the robustness of the response.

In the meta-analysis of piglets born per litter, the total born was 0.49 piglets/litter more for BB than for CON (*p* < 0.001; Table 2). In the overall merged dataset, total piglets born/litter was 0.40 more for BB than for control sows (*p* < 0.005; Table 4). Thus, with two different analyses, BB improved litter size. 

We could only get treatment by parity data from the merged analyses due to limited sample sizes of some parity sows on some farms. The parity by treatment interaction was highly significant (*p* < 0.0001) for total pigs born and total pigs born alive per litter (Table 4). The results show clearly that BB increased litter size in parities 1, 2 and 3, but not in parity 4, 5, and 6 sows. Sows generally increase litter size as they progress from parities 1 through 3. In parities 4 through 6, sows have plateaued in litter size. The data support the hypothesis that BB increased litters size in younger, still-developing sows and when reproductive performance (litter size) was maximized, BB had no effect. Parities 1, 2 and 3 represented 65% of the sows in the herds used. It would be more cost effective to apply BB only on sows of parity 3 or less than to apply it to all sows. However, we have observed significant behavioral stimulation of sows of all parities. The use of BB to induce estrus behaviors is the subject of other studies that are progress. Induction of sexual behaviors by BB use may reduce reliance on live boars who are both expensive to maintain and dangerous to handle.

### 4.3. Potential Mechanism of BOARBETTER^®^ Action

If we better understood pheromonal mechanisms of action, we may be able to apply this technology to improve farm sustainability and reproductive performance. This study was not designed to answer mechanistic questions about boar sexual pheromones.

Historically, we have known about androstenone and androstenol as constituents of boar saliva [7,8]. However, we also understood that these two steroids did not have as powerful of an effect as a live boar on sow sexual behavior [11]. Boar saliva contains three boar-specific volatile molecules: Androstenone, Androstenol and Quinoline [10]. How these volatile molecules activate the brain is the subject of some past work and is a fertile area for future study.

Several mechanisms have been reported to explain how sexual pheromones induce female sexual behaviors and reproductive success. Organs of olfactory perception and hormonal and anatomical changes associated with sexual pheromones are briefly considered here.

Olfactory perception can begin with the binding of a pheromone with a specific olfactory binding protein (OBP; [12]). A specific OBP was recently described for androstenone and androstenol [13]. A specific OBP has not yet been reported for quinoline. Volatile pheromone molecules can bind olfactory and other receptors without an OBP, but because pheromone molecules up-regulate OBP receptors, the olfactory stimulation is greater when combined with OBPs. However, this mechanism also argues that multiple pheromone exposures over time allows for OBP synthesis and perhaps a greater biological effect on behavior and reproduction. In the current study, sows received at least 2 BB sprays on d 4 and 5 after weaning. Multiple days of spraying might keep these OBP upregulated and therefore more effective at stimulating the olfactory system. Experimental evidence shows that Androstenone actives the main olfactory epithelium (MOE) and not the vomeronasal organ (VNO) [14]. Other olfactory organs such as a septal organ of Gruneberg [15] have not been described in the pig. More volatile molecules stimulate the MOE while chemical signals in liquid form are more likely to active the VNO. This finding suggests that the sexual pheromones should be administered as an aerosol rather than a liquid. In this work, BB was applied in a spray to the nose.

Other possible mechanisms that might be used by the pig when BB stimulates the sow include, first that BB may pass from the airway nasal mucosa to the blood and directly to the hypothalamic-pituitary region and the brain [16]. Volatile molecules activate the brain both through binding olfactory receptors and receptors directly in the brain. Second, sexual pheromones are reported to stimulate reproductive hormones [17,18,19]. We do not know if BB causes gonadotropin release in the pig at this time. Finally, because a live, adult boar will cause oxytocin secretion in estrous sows [20] which then causes uterine contractions, it could be that BB may cause oxytocin release and uterine contractions during sexual behaviors. Farm workers reported that when they used BB, they could not pass a post-cervical artificial insemination rod through the cervix due to cervical contractions. Future mechanistic studies will examine if BB causes oxytocin release and uterine and cervical contractions. 

### 4.4. Economics of Reproductive Improvement

Economic drivers of productivity include breeding and farrowing efficiency as well as litter size (total, born alive and weaned), among other variables. More pigs will be produced from a given batch of sows if either farrowing success rates and/or litter size is increased. From the data in the meta-analyses, we report an absolute increase of 3.71% (Table 2; not significant) in FSR rate over all the parities. The relative improvement in FSR was 5.1% (3.71/72.19% FSR for controls). For the first three parities, we report an increase of 5.6% in born alive/litter (*p* < 0.01) for BB-treated sows compared with CON sows (Table 4). Use of BB for parity 1, 2 and 3 sows, on average, increased the total number of pigs born alive/litter in each farrowing batch by 10.7%. Based on a model farm and using our findings, BB increased pigs born alive per sow by 9.3% per batch of sows.

While we did not attempt to measure labor use and savings, some collaborating farms reported labor savings when using BB either as a part of this study or by using BB in other protocols. Some producers used boars to initially check heat in gilts, and then when they bred the gilt or sow a second time, they used BB and no boar. This saves the time that is required to get a boar out of the pen and move him throughout the barn. It also may contribute to reducing the risk of human injury by boars. The use of BB may eventually eliminate the use of live boars in the sow barns, which would save cost and reduce the risk of injury to people.

## 5. Conclusions

This study represents the first published documentation of a novel sexual pheromone in the pig that stimulates reproductive performance. By two methods of data analysis (meta-analysis or a single, large merged dataset), an increase in pigs born alive and total pigs born per litter was observed by use of BB on 12 commercial farm sites. Total piglets born per batch were also increased when BB was used. While we do not fully understand the mechanisms that cause the observed phenomenon, there is an economic incentive to implement this technology. Furthermore, sexual pheromones are considered to be clean, green and ethical technologies [21] that can improve pork production sustainability and reproductive success.

## 6. Patents

Based partly on this work, a patent was issues to McGlone, J.J with the title: Pheromone composition to stimulate reproduction in female suids and methods of use. USA Patent 9480689B1. Publication date 11-17-2017. 

## Figures and Tables

**Table 1:** Definitions of reproductive response measures.

Name	Definition
Weaned sows, number	Number of sows separated from their piglets and moved to the breeding area
Eligible to be bred (ETBB), number	Weaned sows that had an opportunity to be bred (cull sows, for example, removed from analyses)
Breeding rate, %	% of sows bred relative to those ETBB
Conception rate, %	Found pregnant by ultrasound relative to number bred
Conception success rate, %	Found pregnant by ultrasound relative to those ETBB
Farrowing rate, %	Farrowed relative to those bred
Farrowing success rate, %	Farrowed relative to those ETBB
Total Born (TB)	Sum of born alive, stillborn and mummies
Pigs born alive/litter (BA)	Pigs reported born alive per litter
Pigs stillborn/litter (SB)	Pigs reported stillborn per litter
Mummies/litter (MM)	Mummified pigs per litter
Wean to Estrus Interval (WEI)	Number of days from weaning to estrus

**Table 2:** Effects of a novel boar pheromone, BOARBETTER (BB) on measures of reproductive performance of sows ^1^.

Item	N	Control	BB	Difference	Standard Error	*p*-Value
Total sows bred, N	11	1959	2,039	--	--	--
Breeding Rate, %	7	92.44	92.83	0.40	3.21	0.71
Conception Rate, %	6	88.95	91.67	2.71	1.31	0.09
Conception Success Rate, %	6	80.99	83.12	2.13	3.89	0.45
Farrowing Rate, %	9	83.22	86.57	3.35	1.51	0.17
Farrowing Success Rate, %	5	72.29	75.59	3.30	2.22	0.076
Total Born, per litter	10	13.75	14.21	0.46	0.11	0.003
Born alive, per litter	8	12.67	13.01	0.34	0.16	0.059
Stillborn, per litter	8	0.88	0.85	−0.03	0.11	0.81
Mummified, per litter	5	0.17	0.18	0.01	0.03	0.68
Wean to Estrus Interval, WEI	7	4.32	4.40	0.08	0.038	0.07
Gestation Length, GL	7	115.3	115.8	0.44	0.45	0.38

^1^ Data represent an average value per farm site (N). A total of 27,700 sows were represented on study sites ranging from approximately 450–6000 sows per site. The N shown is the number of farm sites (the experimental unit for the meta-analyses) having data for each measure. The *p*-value reflects the meta-analyses *p*-value under the null hypothesis that the difference between Control and BOARBETTER treatments was zero.

**Table 3:** Least squares means (LSM) for Total Born (TB) and Born Alive (BA) by farm site. The *p*-values listed are the within-farm P-values. The averages of these values are presented in Table 2. Data from most farms lack sufficient power to detect differences; see Table 4 for the merged dataset that contains sufficient statistical power.

**Descriptive Variable**			**Total Born**							
			**Control**			**BOARBETTER**				
**Farm Site**	**Parity**	**Weeks**	**Litters N**	**LSM**	**SE**	**Litters N**	**LSM**	**SE**	**Difference**	***p*-Value**
1, 2	1–6	2	78	12.78	0.35	50	13.18	0.44	0.40	0.480
3	1–8	6	72	12.24	0.39	73	12.66	0.39	0.42	0.450
4	1–6	6	34	11.62	0.52	36	12.78	0.51	1.16	0.120
5	--	6	350	13.04	--	383	13.58	--	0.54	--^a^
6	1–7	6	305	14.41	0.19	260	14.22	0.20	−0.19	0.496
7	1–7	6	160	14.64	0.28	162	14.96	0.28	0.32	0.423
8	2–13	6	220	13.68	0.25	200	14.01	0.27	0.33	0.370
9	1–13	7	42	14.45	0.65	51	14.88	0.59	0.43	0.628
10 ^b^	1–8	6	311	14.33	0.21	395	15.18	0.19	0.85	0.003
11	1–6	4	55	15.47	0.48	109	15.74	0.34	0.27	0.647
12	1–8	4	108	15.19	0.37	108	16.10	0.37	0.91	0.084
**Heading Title**			**Born Alive**							
			**Control**			**BOARBETTER**				
**Farm Site**	**Parity**	**Weeks**	**Litters N**	**LSM**	**SE**	**Litters N**	**LSM**	**SE**	**Difference**	***p*-Value**
1, 2	1–6	1	78	12.31	0.34	50	12.58	0.42	0.27	0.614
3	1–8	6	72	11.63	0.37	73	12.08	0.37	0.46	0.380
4	1–6	6	34	11.18	0.52	36	12.31	0.50	1.13	0.122
5	--	6	350	--	--	383	--	--	--	--^a^
6	1–7	6	305	13.31	0.18	260	13.09	0.19	−0.22	0.408
7	1–7	6	160	13.73	0.26	162	13.81	0.26	0.08	0.823
8	2–13	6	220	12.23	0.26	200	12.22	0.26	−0.02	0.960
9	1–13	7	42	11.86	0.60	51	12.92	0.54	1.06	0.191
10 ^b^	1–8	6	311	13.52	0.20	395	14.13	0.18	0.61	0.026
11	1–6	4	55	13.95	0.43	109	14.04	0.30	0.09	0.862
12	1–8	4	108	13.86	0.34	108	14.09	0.35	0.23	0.639

^a^ This farm reported an internal analysis that showed the difference between treatments was significant (*p* < 0.05), but they did not share the raw data, only the overall Least squares means. ^b^ This farm’s data were not included in the overall analyses (Table 2) because this was the only farm that used PCAI instead of conventional AI.

**Table 4:** Results for Total born/litter and Born alive/litter using Mixed Models and parities 1 to 6 for 10 farm sites for Control (CON) and BOARBETTER (BB) treatment groups.

CON					BB			Treatment	*p*-Values from Mixed Model			
Measure	Parity	N	LSMEAN	SE	N	LSMEAN	SE	Difference	Treatment	Parity	TXP	Study
Total pigs born/litter	1	239	13.1	0.25	233	13.9	0.23	0.85	0.01			
	2	349	13.9	0.19	314	14.8	0.20	0.90	0.0006			
	3	307	14.5	0.20	336	15.4	0.19	0.90	0.0005			
	1–3	895	13.8	0.21	883	14.7	0.21	0.88	0.0001			
	4	290	14.5	0.19	313	14.1	0.20	−0.40	0.19			
	5	178	14.5	0.25	184	14.7	0.23	0.20	0.44			
	6	67	14.2	0.38	65	14.1	0.50	−0.10	0.92			
	1–6	1430	14.1	0.11	1445	14.5	0.12	0.40	0.005	0.0001	0.0034	0.0001
Born alive/litter	1	239	12.4	0.24	233	13.1	0.23	0.70	0.02			
	2	349	13.0	0.18	314	13.8	0.19	0.80	0.002			
	3	307	13.5	0.18	336	14.2	0.16	0.70	0.002			
	1–3	895	13.0	0.20	883	13.7	0.19	0.73	0.01			
	4	290	13.4	0.18	313	12.9	0.20	−0.50	0.09			
	5	178	13.4	0.24	184	13.2	0.23	−0.20	0.45			
	6	67	12.7	0.34	65	12.7	0.49	0.00	0.96			
	1–6	1430	13.1	0.10	1445	13.32	0.12	0.22	0.08	0.0001	0.0014	0.0001
